# Supplementary material for: An open label randomized controlled trial of tamoxifen combined with amphotericin B and fluconazole for cryptococcal meningitis
Source: eLife. 2021 Sep 28;10:e68929. doi: 10.7554/eLife.68929 (PMC8547950; doi:10.7554/eLife.68929)
Supplement: Transparent reporting form [file elife-68929-transrepform.docx]

***eLife’s* transparent reporting form**

We encourage authors to provide detailed information *within their submission* to facilitate the interpretation and replication of experiments. Authors can upload supporting documentation to indicate the use of appropriate reporting guidelines for health-related research (see [EQUATOR Network](http://www.equator-network.org/%20)), life science research (see the [BioSharing Information Resource](https://biosharing.org/)), or the [ARRIVE guidelines](http://www.plosbiology.org/article/info:doi/10.1371/journal.pbio.1000412) for reporting work involving animal research. Where applicable, authors should refer to any relevant reporting standards documents in this form.

If you have any questions, please consult our Journal Policies and/or contact us: [editorial@elifesciences.org](mailto:editorial@elifesciences.org).

**Sample-size estimation**

- You should state whether an appropriate sample size was computed when the study was being designed
- You should state the statistical method of sample size computation and any required assumptions
- If no explicit power analysis was used, you should describe how you decided what sample (replicate) size (number) to use

Please outline where this information can be found within the submission (e.g., sections or figure legends), or explain why this information doesn’t apply to your submission:

The study sample size justification is describe on page 7 lines 144 to 149. Further details on the methods used are provided in the published study protocol:

Ngan NTT, Mai NTH, Tung NLN, Lan NPH, Tai LTH, Phu NH, Chau NVV, Binh TQ, Hung LQ, Beardsley J, White N, Lalloo D, Krysan D, Hope W, Geskus R, Wolbers M, Nhat LTH, Thwaites G, Kestelyn E, Day J. A randomized open label trial of tamoxifen combined with amphotericin B and fluconazole for cryptococcal meningitis. *Wellcome Open Res.* 2019;4:8.

**Replicates**

- You should report how often each experiment was performed
- You should include a definition of biological versus technical replication
- The data obtained should be provided and sufficient information should be provided to indicate the number of independent biological and/or technical replicates
- If you encountered any outliers, you should describe how these were handled
- Criteria for exclusion/inclusion of data should be clearly stated
- High-throughput sequence data should be uploaded before submission, with a private link for reviewers provided (these are available from both GEO and ArrayExpress)

Please outline where this information can be found within the submission (e.g., sections or figure legends), or explain why this information doesn’t apply to your submission:

Not applicable

**Statistical reporting**

- Statistical analysis methods should be described and justified
- Raw data should be presented in figures whenever informative to do so (typically when N per group is less than 10)
- For each experiment, you should identify the statistical tests used, exact values of N, definitions of center, methods of multiple test correction, and dispersion and precision measures (e.g., mean, median, SD, SEM, confidence intervals; and, for the major substantive results, a measure of effect size (e.g., Pearson's r, Cohen's d)
- Report exact p-values wherever possible alongside the summary statistics and 95% confidence intervals. These should be reported for all key questions and not only when the p-value is less than 0.05.

Please outline where this information can be found within the submission (e.g., sections or figure legends), or explain why this information doesn’t apply to your submission:

The statistical methods are described in the main manuscript page 8 line 150 to 171. The statistical analysis plan is included in the supplementary data found on pages 33 to 46.

P values and 95CI are presented for the study analyses in the results section.

The statistical code is freely available at <https://doi.org/10.5287/bodleian:XmeOzdR8z>

(For large datasets, or papers with a very large number of statistical tests, you may upload a single table file with tests, Ns, etc., with reference to sections in the manuscript.)

**Group allocation**

- Indicate how samples were allocated into experimental groups (in the case of clinical studies, please specify allocation to treatment method); if randomization was used, please also state if restricted randomization was applied
- Indicate if masking was used during group allocation, data collection and/or data analysis

Please outline where this information can be found within the submission (e.g., sections or figure legends), or explain why this information doesn’t apply to your submission:

The method of randomization is described on lines 115 to 119.

The study is described as open label, although the primary endpoint is microbiological and objective and the technician measuring the microbiological procedures was blinded to the treatment allocation of the patient associated with any sample. The study is therefore protected against bias.

**Additional data files (“source data”)**

- We encourage you to upload relevant additional data files, such as numerical data that are represented as a graph in a figure, or as a summary table
- Where provided, these should be in the most useful format, and they can be uploaded as “Source data” files linked to a main figure or table
- Include model definition files including the full list of parameters used
- Include code used for data analysis (e.g., R, MatLab)
- Avoid stating that data files are “available upon request”

Please indicate the figures or tables for which source data files have been provided:

The clinical trial has been conducted in Vietnam under the Ministry of Health and local Ethical Committee approvals. Requests to share data have to be acknowledged by the local Ethical Committee (and therefore we cannot hand over the data repository or management to an external party). The original de-identified clinical data underlying the study are available by emailing the OUCRU Data Access Committee at DAC@oucru.org or ekestelyn@oucru.org (Head of the Clinical Trials Unit and Data Access Committee Chair). The review procedures (the data sharing policy and the data request form) are available on the OUCRU website at http://www.oucru.org/data-sharing/
